# Integrating Emergency Medical Services Into Health Systems for Continuous and Resilient Care

**DOI:** 10.3389/phrs.2026.1609282

**Published:** 2026-04-22

**Authors:** Gina Marie Gerlach, Sarah Maria Esther Jerjen, Armin Gemperli

**Affiliations:** Faculty of Health Sciences and Medicine, University of Lucerne, Lucerne, Switzerland

**Keywords:** data linkage, emergency medicine, health system resilience, learning health systems, prehospital care

## Abstract

**Objectives:**

Emergency Medical Services (EMS) are central to acute care, disaster response, and public health. Yet prehospital data in many systems remain disconnected from hospital and follow-up outcomes. This paper examines how fragmented, unidirectional data flows limit quality assurance, system learning, and crisis preparedness, using Switzerland as an illustrative case.

**Methods:**

We analyze data flows across the rescue chain based on regulatory context, current handover practices, and international reference models. The analysis is supported by existing registry initiatives and a conceptual systems framework.

**Results:**

Across EMS systems, information is generated in silos and transferred through brief handovers without systematic outcome feedback. Evaluation is therefore reduced to operational metrics such as response times, obscuring the clinical impact of prehospital care. In Switzerland, decentralized governance and the absence of national standards reinforce these dynamics. Existing registries demonstrate that outcome tracking is feasible using minimal standardized datasets.

**Conclusion:**

Bidirectional EMS data exchange is essential to transform linear rescue chains into learning health systems. A national EMS minimum dataset with mandatory reporting and outcome feedback would enable transparency, quality improvement, and resilient emergency care.

Emergency Medical Services (EMS) operate at a crucial intersection of the public health system, connecting patients to immediate medical care during pandemics, disasters, and daily emergencies. Their role extends beyond patient transport to include coordination and communication that can strengthen system preparedness during both routine operations and large-scale crises.

Health system resilience, the ability to maintain essential health services before, during, and after times of crisis, depends on the systematic collection and exchange of reliable health information. This exchange has become increasingly digital, with electronic healthcare data being used to monitor service quality, evaluate interventions, and support evidence-informed decision making. In EMS, transparent and integrated data systems enable assessment, benchmarking, and resource allocation, while also fostering accountability and equity. Cohesive data linkage bridges disaster medicine and public health, supporting both operational preparedness and long-term system improvement.

Switzerland provides an important context to explore the challenges and opportunities for data exchange in a decentralized system. Swiss EMS is regulated at the cantonal level, with no federal requirement governing how providers record or share data with hospitals. Patient handover relies on a short verbal exchange and a brief written report delivered within minutes of arrival [1]. While these practices ensure rapid transfer of essential clinical information, they provide only minimal continuity across the rescue chain.

As illustrated in Figure 1, current data flow across dispatch, EMS, hospital, and follow up care remains siloed. Each stage collects information independently, but there is no mechanism for data integration or outcome feedback. The result is a fragmented, unidirectional system in which valuable clinical information is lost at every transition.

**FIGURE 1:**
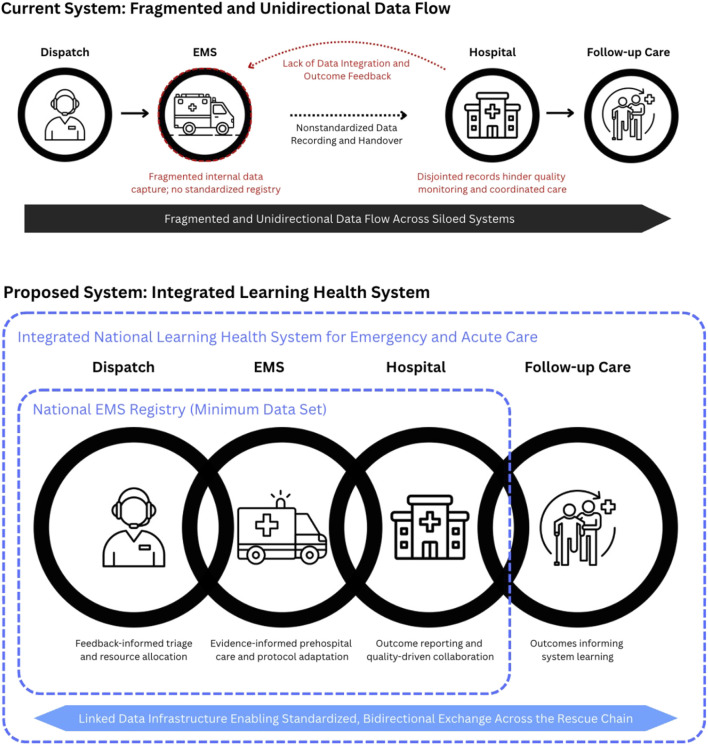
Fragmented versus integrated data flow across the emergency care continuum (Switzerland, 2025). Current and proposed models of information exchange across dispatch, prehospital emergency services, hospital care, and follow-up care. The upper panel illustrates the existing system, characterized by siloed data capture, non-standardized documentation, and unidirectional transfer of information, with no structured integration or outcome feedback across stages of care. The lower panel presents a proposed integrated learning health system based on a national minimum dataset for prehospital emergency care. Linked data infrastructure enables standardized, bidirectional exchange across the rescue chain, supporting feedback-informed dispatch, evidence-based prehospital treatment, outcome reporting, quality-driven collaboration, and continuous system learning.

Without national standards for data collection and exchange, Swiss EMS performance is currently assessed primarily by response times [2]. Timely response is essential, but it does not reflect the clinical value of EMS care. For time-sensitive conditions, early paramedic interventions can lead to faster recognition and treatment [3]. Without linked data, these contributions remain unseen, hindering system evaluation and evolution.

Integrating EMS data with hospital and long-term outcomes is essential for assessing the value of prehospital interventions and the reduction of downstream costs [4]. Without structured, bidirectional data exchange, hospitals, insurers, and regulators are limited in their ability to evaluate triage accuracy, treatment efficacy, and patient outcomes.

EMS evaluation requires a continuous flow of information from dispatch through hospital outcomes, creating a structured feedback loop. This integration allows measurement not only of response times but also of the clinical impact of prehospital interventions. Outcome-linked data can also support the expansion of paramedic roles, including on-scene treatment, palliative care, and prescribing authority, which may relieve pressure on emergency departments and enhance overall system resilience during crises [3].

Hospitals also face disadvantages without prehospital information. Clinicians cannot accurately assess changes in patient condition from EMS pickup to hospital arrival, increasing the risk of triage errors, redundant diagnostics, and delayed intervention [5]. Paramedics rarely receive feedback on diagnoses and patient outcomes, undermining quality assurance and limiting opportunities to refine clinical protocols [6]. These gaps can have serious implications for patient safety, including increased morbidity and mortality.

The lack of bidirectional data exchange does not reflect individual shortcomings. Both paramedics and hospital staff operate under significant clinical and operational pressure, especially during a pandemic or global health crisis, which limits their ability to efficiently link their information without a well-established protocol or electronic system [5]. The systemic absence of data continuity impairs the health system’s ability to accurately assess treatment efficacy, ensure quality control, and manage resources efficiently.

A system of shared outcome reporting would allow healthcare professionals across the rescue chain to evaluate interventions in real-world conditions. Paramedics could learn from hospital diagnoses and outcomes, while hospitals could make more informed decisions based on complete patient trajectories [5, 6].

Recent initiatives show that data linkage in Switzerland is feasible. The Swiss Center for Rescue, Emergency and Disaster Medicine (SCRED) has piloted the Minimal Data Set Switzerland (MiND), collecting standardized emergency department data nationwide [7]. Disease-specific registries such as SWISSRECA for out-of-hospital cardiac arrest [8] and the Swiss Trauma Registry for severe trauma demonstrate that outcome tracking is possible even without a centralized electronic health record [9].

Other countries provide examples that Switzerland can adapt. The U.S. National EMS Information System (NEMSIS) offers a standard for prehospital data collection [10]. This case shows that bidirectional data can be achieved through minimal standardized datasets, legal mandates, and targeted governance rather than complete electronic record reform.

Figure 1 illustrates how a national EMS minimum dataset can transform the current linear pathway into an integrated learning system, enabling feedback-informed triage, evidence-based prehospital care, quality-driven care coordination, and outcome data that informs continuous system learning.

The core challenge for Swiss EMS is regulatory rather than technological. Without tools to facilitate transparent, bidirectional data sharing, Switzerland’s health system resiliency is limited. Transparency provides stakeholders with information to make well-informed decisions. Clinical transparency involves accessible documentation of diagnoses, treatments, and patient trajectories. Financial transparency clarifies billing, cost-sharing and reimbursement. Operational transparency refers to the infrastructure that enables financial and clinical transparency, including standardized coding, triage, and benchmarking systems. Coordinated EMS data allow stakeholders to make decisions that improve the quality and efficiency of care.

The absence of transparency reflects historical design rather than negligence. As care complexity increases and outcomes become more scrutinized, structural challenges must be addressed. Cantonal regulators, EMS providers, and hospital administrators each have a role in establishing national coding standards, defining a minimal dataset, and enabling basic outcome tracking for key emergency conditions.

The financing divide between EMS and hospital care must be addressed. From the patient’s perspective, an emergency is a continuous event, but billing occurs independently across providers. Current financing models separate prehospital and hospital care by reimbursing EMS for transportation rather than clinical contributions. Financing models that span the full continuum of care, like episode-based or bundled payments, would make linked data exchange a financial necessity, supporting a more coherent, resilient, and patient friendly health system.

To prepare for future global health crises, it is recommended for Switzerland to establish a national EMS registry with mandatory reporting of a standardized minimum dataset, that is linked across dispatch, EMS, and hospital outcomes. This registry would create a foundation for transparency, quality improvement, system monitoring, and research. Participation includes bidirectional feedback loops to ensure both EMS providers and hospitals gain meaningful insights into the quality of their care.

Transparent EMS data exchange is a prerequisite for integrated epidemic and disaster preparedness. EMS operates at the convergence of acute care, disaster response, and public health surveillance. Without linked prehospital and hospital outcomes, meaningful integration cannot occur. The COVID-19 pandemic has shown that resilient health systems rely on real-time performance monitoring, protocol adaptation, and resource allocation. A transparent, standardized, and bidirectional EMS–hospital data framework would enable Switzerland to move from reactive crisis response to proactive system learning. By measuring patient outcomes, Swiss EMS can become a cornerstone of national health resilience and a model of integration between public health and disaster medicine.
